# Visible-Light-Driven Photocatalytic Activity of Magnetic BiOBr/SrFe_12_O_19_ Nanosheets

**DOI:** 10.3390/nano9050735

**Published:** 2019-05-13

**Authors:** Taiping Xie, Jiao Hu, Jun Yang, Chenglun Liu, Longjun Xu, Jiankang Wang, Yuan Peng, Songli Liu, Xiuyu Yin, Yuanzhen Lu

**Affiliations:** 1 State Key Laboratory of Coal Mine Disaster Dynamics and Control, Chongqing University, Chongqing 400044, China; deartaiping@163.com; 2 Chongqing Key Laboratory of Extraordinary Bond Engineering and Advanced Materials Technology (EBEAM), Yangtze Normal University, Chongqing 408100, China; wjkwjk074478@163.com (J.W.); pengyuan1030@sina.com (Y.P.); 3 College of Chemistry and Chemical Engineering, Chongqing University, Chongqing 401331, China; hujiao666@163.com (J.H.); xinyuyin@163.com (X.Y.); yuanzhenlu@163.com (Y.L.); 4 College of Materials and Chemical Engineering, Chongqing University of Arts and Sciences, Yongchuan 402160, China; yang_jun_yang_jun@163.com

**Keywords:** BiOBr/SrFe_12_O_19_, photocatalyst, Rhodamine B, photodegradation rate, magnetic photocatalyst

## Abstract

Magnetic BiOBr/SrFe_12_O_19_ nanosheets were successfully synthesized using the hydrothermal method. The as-prepared samples were characterized by X-ray diffraction (XRD), scanning electron microscope (SEM), transmission electron microscope (TEM), and UV-visible diffused reflectance spectra (UV-DRS), and the magnetic properties were tested using a vibration sample magnetometer (VSM). The as-produced composite with an irregular flaky-shaped aggregate possesses a good anti-demagnetization ability (Hc = 861.04 G) and a high photocatalytic efficiency. Under visible light (λ > 420 nm) and UV light-emitting diode (LED) irradiation, the photodegradation rates of Rhodamine B (RhB) using BiOBr/SrFe_12_O_19_ (5 wt %) (BOB/SFO-5) after 30 min of reaction were 97% and 98%, respectively, which were higher than that using BiOBr (87%). The degradation rate of RhB using the recovered BiOBr/5 wt % SrFe_12_O_19_ (marked as BOB/SFO-5) was still more than 85% in the fifth cycle, indicating the high stability of the composite catalyst. Meanwhile, after five cycles, the magnetic properties were still as stable as before. The radical-capture experiments proved that superoxide radicals and holes were main active species in the photocatalytic degradation of RhB.

## 1. Introduction

Throughout the last few years, semiconductor photocatalysts, which can utilize the clean, renewable, and most accessible solar energy, have attracted more and more attention in the material science field [1,2]. The photocatalytic degradation of organic pollutants under sunlight irradiation possesses promising applications in water pollutant control. Composite photocatalytic materials are receiving considerable attraction owing to their excellent photocatalytic activity and stability [3,4,5,6,7,8].

Bismuth oxyhalide (BiOX; X = Cl, Br, and I) has been widely studied because of its narrow band gap (E_g_ = 1.7–3.4 eV) [9,10,11]. In particular, BiOBr, with an intrinsic lamellar structure and outstanding photocatalytic property, is one of the most promising photocatalysts. However, the E_g_ of BiOBr is 2.8 eV, leading to a low absorption ability of visible light and a high recombination rate of photo-induced electron-hole pairs [12,13]. There are reports on exploring strategies for the improvement of visible light absorption and enhancement of the charge separation efficiency of BiOBr [14,15,16,17]. However, the problem of a low recycling rate after the photocatalytic reaction still exists. The recovered method using an external magnet is a quick and low-cost approach [18], if the catalyst materials hold magnetization.

The development research of magnetic photocatalysts (e.g., Mn_x_Zn_1-x_Fe_2_O_4_/βBi_2_O_3_, Fe_3_O_4_/BiOCl, BiOBr/ZnFe_2_O_4_, BiOBr/CoFe_2_O_4_, and Ag/AgCl/CoFe_2_O_4_) [19,20,21,22,23,24] is interesting, because of its easier recyclable and repeatable use. It is worth noting that hard-magnetic strontium ferrite (SrFe_12_O_19_) has a large saturation magnetization, superior coercivity, good chemical stability, and corrosion resistivity [25]. It was reported that SrFe_12_O_19_ inhibited facet growth [1] and the selective exposure of bismuth-based semiconductor photocatalyst [26]. Consequently, visible light absorption was enhanced by compositing SrFe_12_O_19_, involving the low band gap energy and resulting in quick charge separation and increased photocatalytic ability.

The photocatalytic degradation of organic contaminants under sunlight is a bright application prospect in pollution control engineering. However, sunlight is a hot light and easily causes a series of side reactions in the process of the photocatalysis reaction. In our previous studies [19,25], magnetic photocatalysts were prepared by a dip-calcination method. It is noted that light emitting diodes (LEDs) with little heat and good linearity are superior to conventional light sources or to sunlight. Thus, LEDs have been widely used in hydrogen production and organic synthesis over semiconductor photocatalysts [27,28,29,30]. The outstanding advantages of LED light are their long-life time and high controllability in the reaction progress.

In this work, magnetic nanosheet BiOBr/SrFe_12_O_19_ (BOB/SFO-5) was prepared using a reasonable fabrication method, and the corresponding photocatalytic property was investigated under visible light and UV-LED (390–410 nm). Further insight into the magnetization and repeatable rate, and the photocatalytic mechanism were researched with different kinds of tests. The photocatalytic mechanism assisted by a magnetic field has been further discussed in this research. These results provide important concepts concerning the synthesis method, and pave the way for the industrial application of magnetic photocatalysts.

## 2. Experimental Section

All reagents were of analytical grade and were used directly, without further purification. The water was deionized throughout all the experiments.

### 2.1. Preparation of BiOBr/SrFe_12_O_19_

Solution A consisted of 5 mmol of Bi(NO_3_)_3_·5H_2_O and 762 mg of SrFe_12_O_19_ (prepared by a hydrothermal method [31]) dissolved in 20 mL of deionized water. Afterwards, 15 mL of NaBr (5 mmol) was slowly dipped into solution A, and the formed suspension was transferred into a 50 mL Teflon-lined stainless-steel autoclave and heated to 160 °C for 12 h. Subsequently, the solid was separated and washed several times with deionized water and absolute alcohol, alternately. Then, the washed solid was dried at 60 °C for 12 h, and the BiOBr/5 wt % SrFe_12_O_19_ (marked as BOB/SFO-5) was obtained. A series of BiOBr-based composite products, including 0 wt %, 3 wt %, 7 wt %, 10 wt %, and 15 wt % SrFe_12_O_19_, were prepared using the same process, and the gained composites were noted as BOB, BOB/SFO-3, BOB/SFO-7, BOB/SFO-10, and BOB/SFO-15, respectively.

### 2.2. Material Characterization

Phase identification via X-ray diffraction (XRD) was conducted on an X-ray diffractometer (Bruker Advance D8, Bruker, Germany). The average crystallite sizes of the samples were calculated from the XRD peak (102) using the classical Scherrer equation, D_hkl_ = Kλ/βcosθ, where D_hkl_ is the particle diameter (nm), K is the constant (0.943), λ is the X-ray wavelength (0.15405 nm), β is the half-maximum line width, and θ is the diffraction angle. The microstructural morphology was observed by scanning electron microscopy (SEM; EVO-LS15X, ZEISS, Oberkochen, Germany) and high-resolution transmission electron microscopy (HR-TEM). Energy dispersive analysis (EDS) systems were used to verify the element constituents. A vibrating sample magnetometer (VSM; Lakeshore 7410, Lake Shore, Carson, USA) was employed to determine the magnetization. The UV-VIS diffuse reflectance spectra (DRS) were measured using a UV-VIS spectrophotometer (TU1901, Beijing Purkinje General Instrument Co. ltd, Beijing, China).

### 2.3. Determination of Photocatalytic Property

The photocatalytic performance of the BOB/SFOs was evaluated using the Rhodamine B (RhB) photodegradation under visible light (λ > 420 nm) and UV light-emitting diode (LED) irradiation. The visible light was obtained using a 300 W Xenon lamp with a UV cut-off filter (CEL-HXF300, AULTT, Beijing, China), and the UV light was acquired by the LED under cool light (λ = 390–410 nm; power = 122 μW). In addition, the distance between the light source and the reactor was 10 cm. A 100 mL RhB solution (10 mg/L) and 50 mg catalyst were added into a quartz container and stirred for 30 min in the dark, so that the adsorption equilibrium was reached. The irradiation time was set as 30 min in the process of continuous stirring. Then, 3 mL of the RhB solution was sampled in the setting time interval. The photocatalytic degradation process of the RhB was monitored by measuring the characteristic absorption at 554 nm with a UV-VIS spectrophotometer. Composite catalysts were separated and recovered with an external magnet.

The photocatalytic mechanism was probed using active species trapping experiments. The degradation rates of the RhB over the photocatalyst were measured in active species scavenger agents, including the hydroxyl radical scavenger of isopropanol (IPA), the hole scavenger of disodium ethylenediaminetetra acetic acid (Na_2_-EDTA), and the superoxide radical scavenger of 1,4-benzoquinone (BZQ), respectively.

## 3. Results and Discussion

A primary analysis of the photodegradation revealed that BOB/SFO-5 was the most efficient in the RhB degradation under UV irradiation.

### 3.1. Microstructure and Pore Characteristics

**Figure 1:**
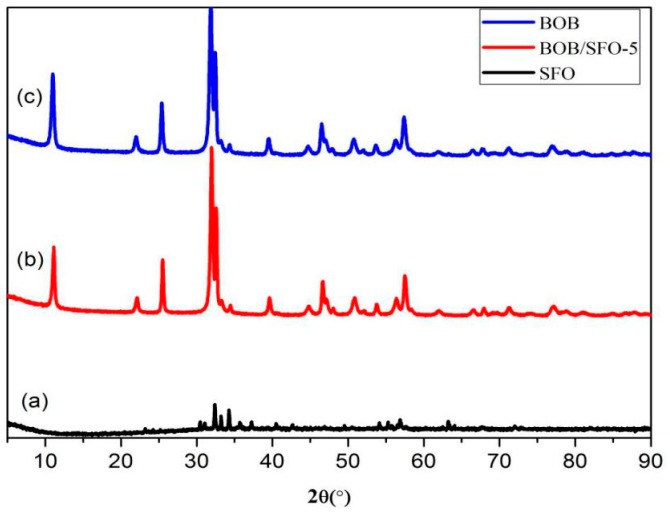
X-ray diffraction (XRD) patterns of pure (**a**) SrFe_12_O_19_ (SFO), (**b**) BiOBr (BOB), and (**c**) BiOBr/SrFe_12_O_19_ (5 wt %) (BOB/SFO-5).

Figure 1 shows the XRD patterns of SrFe_12_O_19_, BiOBr, and BOB/SFO-5. As we can see from Figure 1a, the pattern of SrFe_12_O_19_ revealed a hexagonal primitive crystal structure (JCPDS card no. 33-1340) [32]. This was a member of the space group, P_63_/mmc, and the lattice parameters were a = b = 5.8868 Ǻ and c = 23.037 Å. The main diffraction peaks of BiOBr (Figure 1c) were located at 2*θ* = 10.975°, 21.979°, 25.390°, 31.851°, 32.432°, and 57.403°, which matched with the (001), (002), (101), (102), (110), and (212) crystal planes, respectively (JCPDS card no. 85-0862; space group P_4_/nmm; a = b = 3.92 Å; c = 8.11 Å). For BOB/SFO-5 (Figure 1b), the diffraction peak of BiOBr was relatively strong and that of SrFe_12_O_19_ was relatively weak, and the (002), (110), (111), and (114) peaks of BiOBr overlapped with the (103), (107), (114), and (304) diffraction peaks of SrFe_12_O_19_, respectively. Thus, the characteristic peaks of SrFe_12_O_19_ in the pattern of BOB/SFO-5 were not distinct. 

It is worthwhile mentioning that the amount of magnetic matrix (5 wt %) was relatively low, and no impurity phase was found in BiOBr/SrFe_12_O_19_, confirming that there is no appreciable decomposition and perceptible chemical reaction between BiOBr and SrFe_12_O_19_.

The average crystallite sizes of BiOBr and BiOBr/SrFe_12_O_19_ calculated by the Scherrer equation were 24.26 nm and 19.77 nm, respectively. The small size of BiOBr/SrFe_12_O_19_ was possibly due to the growth inhibition of BiOBr by SrFe_12_O_19_ [27].

**Figure 2:**
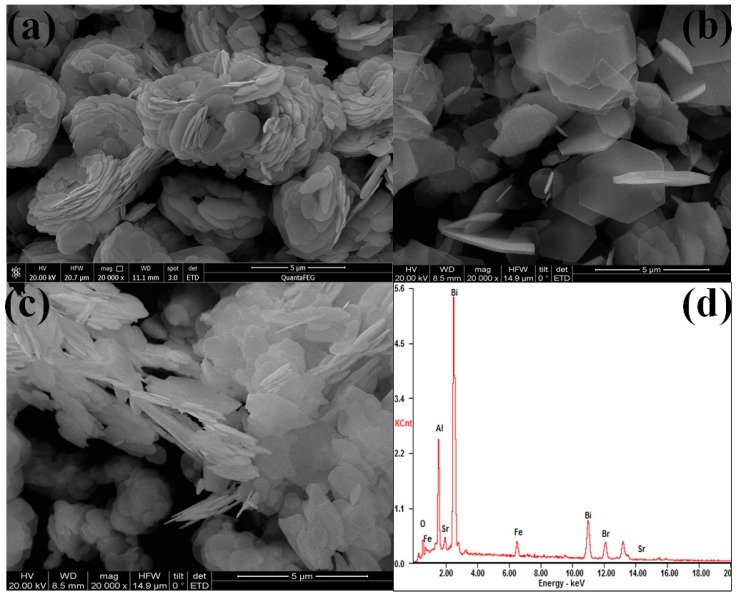
Scanning electron microscope (SEM) images of pure(**a**) BOB, (**b**) SFO, and (**c**) BOB/SFO-5; (**d**) energy dispersive analysis (EDS) of BOB/SFO-5.

The morphological characteristics (Figure 2) of SrFe_12_O_19_, BiOBr, and BOB/SFO-5 were investigated using SEM. As shown in Figure 2a, the SrFe_12_O_19_ is a hexagonal-shape, which is consistent with the literature [26]. Figure 2b shows that the BiOBr nanosheets assembled as “petals” and formed a flower-like microstructure, which could provide more active sites for the adsorption of organic pollutants and help to enhance the photocatalytic activity for the pollutants’ decomposition [33]. As for BOB/SFO-5 (Figure 2c), SrFe_12_O_19_ nanosheets were inserted into a BiOBr flower-like shape, which indicated that the irregularly flaky-shape aggregated on the surface of BiOBr. The intimate interaction between BiOBr and SrFe_12_O_19_ might contribute to the improvement of the electron transfer and separation capacity in the photocatalysis process. Furthermore, the EDS spectra of BOB/SFO-5 is shown in Figure 2d, confirming the existence of the Sr, Fe, O, Br, and Bi elements.

**Figure 3:**
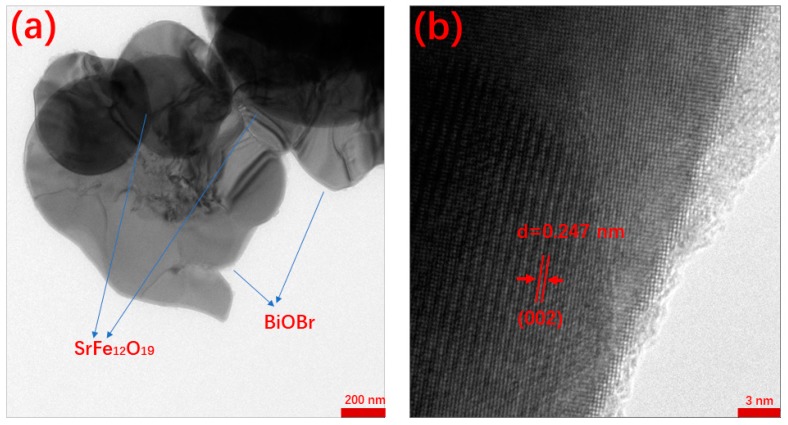
(**a**) Transmission electron microscope (TEM) and (**b**) high-resolution transmission electron microscopy (HR-TEM) of BOB/SFO-5.

The TEM image of BOB/SFO-5 is given in Figure 3a, where the nanosheet-shaped structure is further proven. Figure 3b shows the HR-TEM image, which demonstrates that the nanosheet was well crystalized, and the interplanar of the (002) plane of the monoclinic BiOBr is 0.247 nm. Furthermore, BiOBr is a p-type semiconductor material and SrFe_12_O_19_ is an n-type semiconductor material, leading to a p–n heterojunction structure of BOB/SFO. In this way, the magnetic composite might hold a high photogenerated charge separation ability.

In addition, the adsorption–desorption isotherms and the pore size distribution curves for BOB/SOF-5 were tested using an ASAP 2010 instrument (micromeritics with a surface area deviation of 1%) (ASAP-2010, Micromeritics, Norcross, GA, USA). The results are shown in Figure S1. The most probable pore radius was 7.7 nm, which indicates that the composite belongs to a mesopore material that could provide more active sites for photocatalytic experiments.

### 3.2. Optical Properties

**Figure 4:**
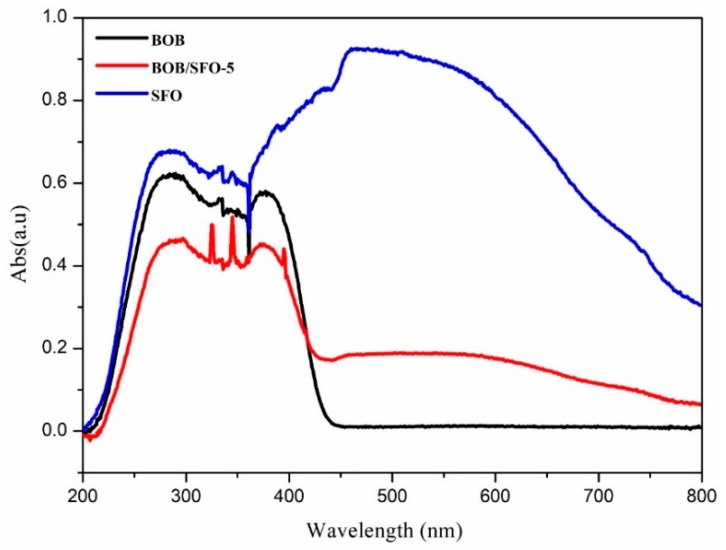
UV-VIS diffuses reflectance spectra of BOB, SFO, and BOB/SFO-5.

The optical absorption property plays a critical role in the photocatalytic performance. The UV-VIS DRS spectra of the BiOBr and BOB/SFO-5 composite are shown in Figure 4. There is an obvious difference in the light absorption abilities of the two samples. It is clear that both the pure BiOBr and the composite present a strong absorption in the UV light region (wavelength of 200–400 nm). However, it is worth noting that the composite showed a strong absorption in a wide wavelength range from UV to visible light, compared with that of pure BiOBr. Therefore, the visible light absorption ability of BiOBr was enhanced for the BOB/SFO-5 composite, and it can be adopted as a visible light-driven catalyst. According to the formula, $\alpha h\upsilon =A(h\upsilon -E_{g}){}^{n/2}$, the band gap energy (E_g_) of BOB, SFO, and BOB/SFO-5 were estimated from the (αhυ)^n/2^ versus photo energy (hυ). Generally speaking, the E_g_ values can be obtained from the intercept of the tangent to the absorption curves. The estimated E_g_ values of BOB, SFO, and BOB/SFO-5 are 2.80 eV, 1.86 eV, and 2.67 eV, respectively. In addition, the estimated E_g_ values are listed in Table S1. Obviously, the decrease in the E_g_ value is due to the introduction of SrFe_12_O_19_. The results show that a new defective energy level formed between the band gap of BiOBr and SrFe_12_O_19_.

### 3.3. Magnetic Property

**Figure 5:**
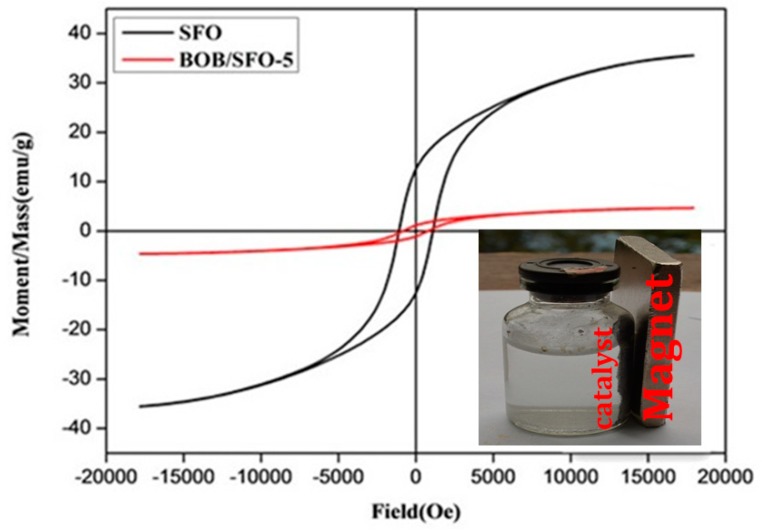
Magnetic hysteresis loops of BOB/SFO-5. Inset shows the magnetization of BOB/SFO-5 in a magnetic field.

The magnetic hysteresis loops of BOB/SFO-5 and SrFe_12_O_19_ are depicted in Figure 5, revealing the typical feature of hard-magnetic materials [34]. Table S2 lists the magnetic parameters of the samples. The saturation magnetization (Ms) of BOB/SFO-5 was 11.3% c.a. lower than that of SrFe_12_O_19_, and the remnant magnetization (Mr) was significantly cut down because of the decreased amount of magnetic component (SrFe_12_O_19_) per gram. Furthermore, it was confirmed that the BiOBr/SrFe_12_O_19_ was successfully synthesized without any negative changes to the magnetization. The results show that the BiOBr/SrFe_12_O_19_ microspheres displayed a good magnetic performance and were easily separated and recovered after the photocatalytic reaction (Figure 4 inset).

### 3.4. Photocatalytic Property

**Figure 6:**
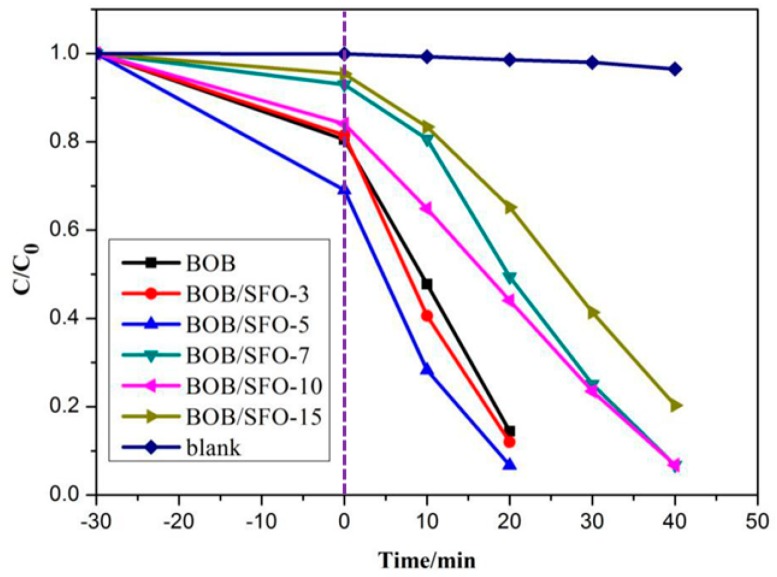
Photocatalytic degradation rate in pure BOB and composites of different amounts of SFO.

The photocatalytic performances of the as-prepared photocatalysts were investigated with RhB photodegradation. Figure S2 shows the absorption curves of RhB in BiOBr/SrFe_12_O_19_ at different times under visible light (λ > 420 nm) irradiation. The key peak intensity (λ_max_ = 554 nm) of RhB declined gradually and reached zero after 30 min of the reaction, indicating the complete photodegradation of RhB using BOB/SFO-5 after only 30 min. Thus, the optimization reaction time was set at 30 min. For more details, a series of tests are shown in Figure 6 that were employed to test the effect of the matrix amount on the degradation rate. In view of the low degradation rate in the blank test, it was necessary to boost the degradation rate with suitable catalysts. Further insights demonstrated that the photocatalytic activity of BOB/SFO-5 was indeed higher than that of pure BiOBr. Under visible light (λ > 420 nm) irradiation, the photodegradation rate of RhB using BOB/SFO-5 after 30 min of reaction could reach 97%, which was higher than that using BiOBr (86%). Compared with the results in the literature [26], the photodegradation rate was obviously enhanced because of a UV cut-off filter that was equipped to ensure a single visible light without UV light from the Xe light resource. When the Xe lamp was replaced with a UV LED (cool light), the degradation rate of the RhB was 98% and 87%, respectively, under the same condition. The results above revealed that BOB/SFO-5 was extremely efficient in RhB degradation under visible light and UV LED irradiation. It can be deduced that there was a strong interaction between the BiOBr nanoparticles and SrFe_12_O_19_ in the hydrothermal reaction, which played a crucial role in the transfer and separation of photogenerated carriers. Particularly, SrFe_12_O_19_ provided a high mobility of the charge carrier through the longitudinal direction, owing to the intimate interaction between BiOBr and SrFe_12_O_19_. Thus, the photogenerated electrons can easily be transferred to the BiOBr nanoflakes’ moiety, leading to the efficient separation and slow recombination of electron-hole pairs. Therefore, BOB/SFO-5 possesses an excellent visible-light-driven catalytic property and can be utilized in subsequent experiments. 

In fact, we investigated some of the references reported in the past 10 years in order to compare them with different photocatalysts under visible light irradiation in terms of the RhB photodegradation ratio. The results are listed in Table S3. It was worth mentioning that the photocatalytic activity of BOB/SFO-5 for RhB photodegradation under UV LED irradiation was outstanding. To the best of our knowledge, the photocatalytic rate reached 97% after only 30 min of photocatalytic reaction under UV LED irradiation, the efficiency of which was superior to that of the existing literature reports.

### 3.5. Stability and Recycle Property

**Figure 7:**
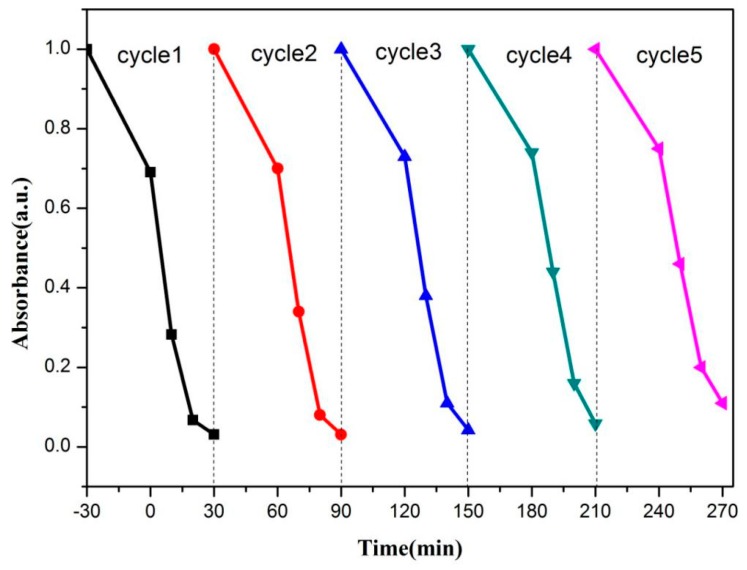
Cycling tests of photocatalytic degradation Rhodamine B (RhB) in BOB/SFO-5 under visible light irradiation.

**Figure 8:**
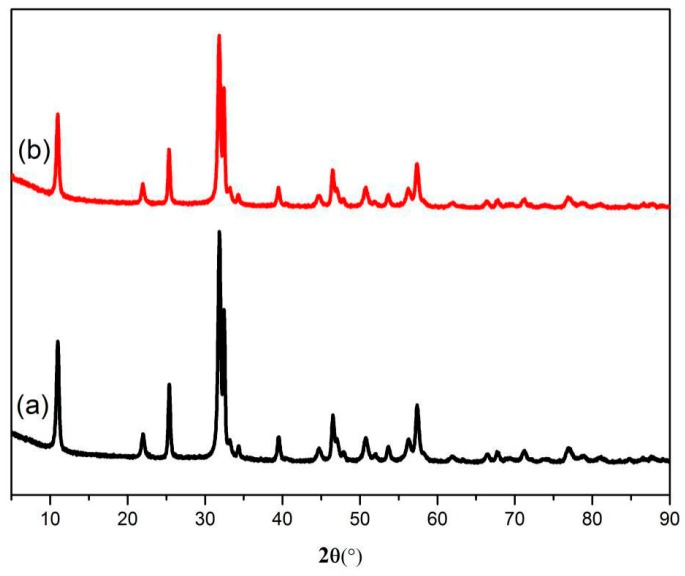
XRD patterns of (**a**) BOB/SFO-5 and (**b**) recovered BOB/SFO-5.

**Figure 9:**
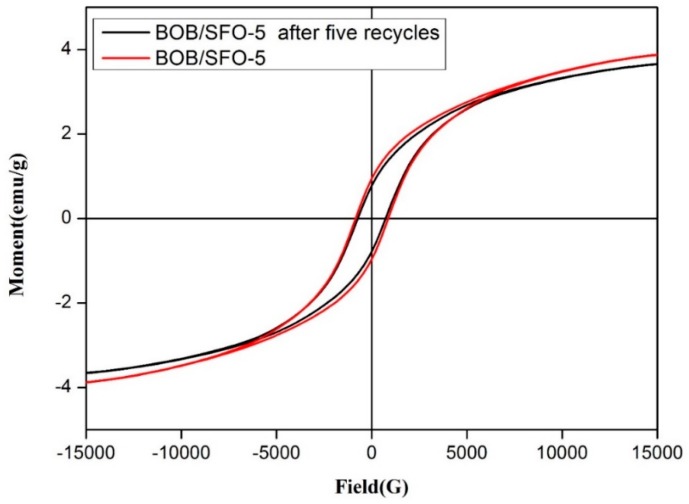
The magnetic hysteresis loops of the BOB/SFO-5 composites.

The photocatalytic stability of BiOBr/SrFe_12_O_19_ was confirmed by the recycling tests. The irradiation time of the four subsequent times cycles was set as 60 so that the degradation rate change was clearly detected. The results are shown in Figure 7. The degradation rate of the RhB in the recovered catalyst was above 85% in the fifth cycle. After five cycles, the BOB/SFO-5 was separated and recovered with an external magnet, and the XRD patterns of the samples were collected, as shown in Figure 8. The same intrinsic crystal structure of the BOB/SFO-5 and recovered BOB/SFO-5 was further confirmed. In addition, the magnetic property of the recovered BOB/SFO-5 was examined and is shown in Figure 9. The Ms, Mr, and Hc of the recovered sample were 4.38 emu/g, 1.18 emu/g, and 861.04 G, respectively. Compared with the parameters of the original BOB/SFO-5, there was almost no decrease of the Ms and coercivity, while the Mr appeared to have little increase. The results indicated that BOB/SFO-5 possessed rather stable magnetic properties. The above achievements illustrated that BOB/SFO-5 exhibited a good stability and high repeatable ability, which overcame the recycling difficulty in the photocatalytic application.

### 3.6. Photocatalytic Mechanism

**Figure 10:**
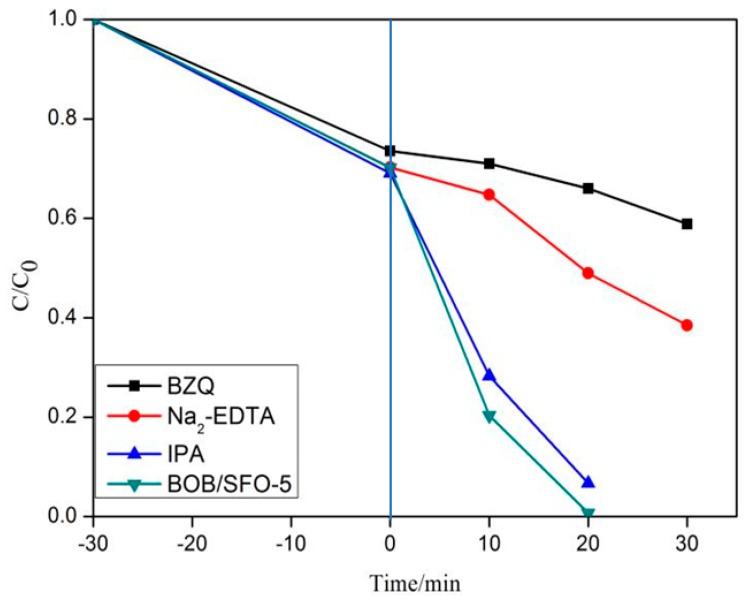
Degradation rates of RhB with BOB/SFO-5 in different hole-radical scavengers (1.0 mmol/L). BZQ—1,4-benzoquinone; Na_2_-EDTA—disodium ethylenediaminetetra acetic acid; IPA—isopropanol.

**Figure 11:**
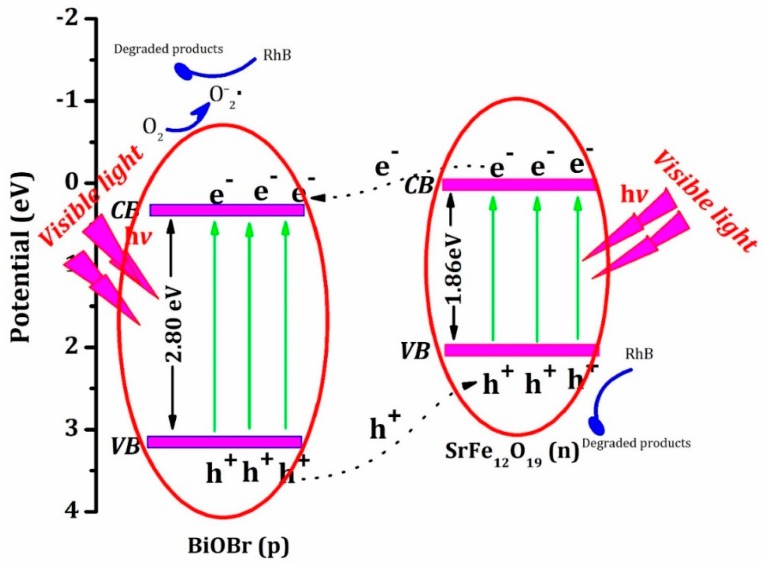
Photocatalytic mechanism scheme of BOB-SFO-5 under visible light irradiation.

It was important to detect the main active species in the photocatalytic process in order to know how to improve the photocatalytic property. Figure 10 shows the RhB degradation rates over BOB/SFO-5 under different active radical species scavengers. It can be seen from Figure 10 that the introduction of the superoxide radical (O_2_^−^) scavenger, BZQ, caused degradation rate declination, namely, the photocatalytic rate was directly proportional to the amount of O_2_^−^, proving the dominant role of O_2_^−^ in the photocatalytic process, which was identified by the EPR spectra for DMPO, and O_2_^−^ acted as the most active species [33,34]. The RhB degradation rates in the hole (h^+^) scavenger, Na_2_-EDTA, were slightly larger than those in BZQ. It can be deduced that the effect of the photo-generated holes was lower than that of O_2_^−^, although the hole was also one of the main oxidation species. Furthermore, the degradation rate of the hydroxyl radical (·OH) scavenger, IPA, was similar to that of BZQ, indicating the smaller difference for the amount change of ·OH. The results above manifest that the photocatalytic reaction of BiOBr/SrFe_12_O_19_ was mainly affected by the O_2_^−^ radicals though the presence of holes. Figure 11 shows the photocatalytic mechanism scheme of BiOBr/SrFe_12_O_19_ under visible light or LED irradiation. 

It is known that BiOBr and SrFe_12_O_19_ are the p-type and n-type semiconductors, respectively. The intimate contact effect of BiOBr/SrFe_12_O_19_ is analogous to a p–n heterojunction structure in the photocatalytic performance, which was absent in the single BiOBr. In addition, the conduction band (CB) and valence band (VB) potentials of the p-type semiconductor, BiOBr, were 0.30 and 3.10 eV [35,36], respectively. Meanwhile, the CB and VB of n-type SrFe_12_O_19_ were 0.20 and 2.06 eV, respectively [37,38]. Thus, the photogenerated electrons in SrFe_12_O_19_ easily transferred to BiOBr, because of the lower CB potential position of BiOBr than that of SrFe_12_O_19_, and the VB holes in BiOBr spontaneously moved to SrFe_12_O_19_ (shown in Equation (1)). The transfer of the electron and hole effectively reduced the charge recombination rate, and resulted in a superior photocatalytic performance. Moreover, the photogenerated electrons inhibited the formation of·HO_2_ (shown as Equations (2) and (3)). As a result, O_2_^−^ directly caused RhB molecule oxidation in the simultaneous reaction of holes in Equation (4).

(1) $$\mathrm{BiOBr}/{\mathrm{SrFe}}_{12}O_{19}+h\nu (\geq \mathrm{Eg})\to \mathrm{BiOBr}(e^{-})/{\mathrm{SrFe}}_{12}O_{19}(h^{+}),$$

(2) $$\mathrm{BiOBr}(e^{-})+O_{2}\to \mathrm{BiOBr}+O_{2}\cdot^{-},$$

(3) $$O_{2}\cdot^{-}+H_{2}O\to \cdot {\mathrm{HO}}_{2}+{\mathrm{OH}}^{-},$$

(4) $${\mathrm{SrFe}}_{12}O_{19}(h^{+}),O_{2}\cdot^{-}+\mathrm{RhB}\to \mathrm{deradation}\;\mathrm{product}.$$

According to the previous study using GC-MS and FT-IR measurements to discuss the mechanism of RhB oxidation [39,40,41], according to the processing steps in the photocatalytic reaction, the RhB was changed from the primary complex macromolecular organic matter to several small organic matters, like phthalic acid [39]. Finally, all of the matter would be degraded into CO_2_ and H_2_O, achieving complete mineralization [40,41]. So, the phenomenon where the blue shift (Figure S2) of the absorption peaks in the ultraviolet-visible absorption spectra appeared in the photodegradation process was easier to understand.

## 4. Conclusions

(1) Nanosheet and irregular flaky-shaped BOB/SrFe_12_O_19_ was successfully prepared by a facile hydrothermal method. The as-prepared composite presented good magnetic properties (Hc = 861.04 G). The recycling experiments proved that the degradation rate of the RhB of the recovered photocatalyst still maintained at 85% in the fifth cycle. The magnetic photocatalyst was conducive to separation and reuse using an external magnet, so that the recycling problem in the industrial application was easily solved.
(2) The excellent visible-light-driven catalytic activity of BOB/SFO-5 was investigated. An Xe lamp (λ > 420 nm) equipped with a UV cut-off filter used as the light resource provided the visible light without UV light. An LED was employed as a cool light resource. The photodegradation rate of the RhB over the BOB/SFO-5 was 97% and 98% at 30 min, which was higher than that (86% and 87%, respectively) over BiOBr.
(3) The superoxide radicals and holes were demonstrated to illustrate the main active species in the photocatalytic reaction of BiOBr/SrFe_12_O_19_. The stable magnetic property of the composite photocatalyst prompted visible light absorption and utilization, producing photogenerated e^−^ and h^+^. SrFe_12_O_19_ further strengthened the light response and enhanced the photocatalytic property of BiOBr by means of absorbing a great number of photons.

## Supplementary Materials

The following are available online at https://www.mdpi.com/2079-4991/9/5/735/s1, Figure S1: Adsorption–desorption isotherms and the pore size distribution curves (Inset) for BOB/SOF-5; Figure S2: Absorption curves of RhB with BOB/SFO-5 under visible light irradiation; Table S1: The estimated Eg values of the as-prepared samples; Table S2: Magnetic parameters of the as-synthesized samples; Table S3: Comparison of photodegradation ratio using different photocatalyst under visible light irradiation reported in the past ten years.

## References

[1] Chatterjee D., Dasgupta S. (2005). Visible light induced photocatalytic degradation of organic pollutants. J. Photochem. Photobiol. C Photochem. Rev..

[2] Zhao J., Wu T., Wu K., Oikawa K., Hidaka H., Serpone N. (1998). Photoassisted Degradation of Dye Pollutants. 3. Degradation of the Cationic Dye Rhodamine B in Aqueous Anionic Surfactant/TiO_2_ Dispersions under Visible Light Irradiation: Evidence for the Need of Substrate Adsorption on TiO_2_ Particles. Environ. Sci. Technol..

[3] Yang X., Cao C., Erickson L., Hohn K., Maghirang R., Klabunde K. (2009). Photo-catalytic degradation of Rhodamine B on C-, S-, N-, and Fe-doped TiO_2_ under visible-light irradiation. Appl. Catal. B Environ..

[4] Rauf M.A., Ashraf S.S. (2009). Fundamental principles and application of heterogeneous photocatalytic degradation of dyes in solution. Chem. Eng. J..

[5] Neppolian B., Choi H.C., Sakthivel S., Arabindoo B., Murugesan V. (2002). Solar/UV-induced photocatalytic degradation of three commercial textile dyes. J. Hazard. Mater..

[6] Zhang J., Xiong Z., Zhao X.S. (2011). Graphene-metal-oxide composites for the degradation of dyes under visible light irradiation. J. Mater. Chem..

[7] Zaccariello G., Moretti E., Storaro L., Riello P., Canton P., Gombac V., Montini T., Rodriguez-Castellon E., Benedetti A. (2014). TiO_2_-mesoporous silica nanocomposites: Cooperative effect in the photocatalytic degradation of dyes and drugs. RSC Adv..

[8] Tian G., Chen Y., Zhou W., Pan K., Dong Y., Tian C., Fu H. (2011). Facile solvothermal synthesis of hierarchical flower-like Bi_2_MoO_6_ hollow spheres as high performance visible-light driven photocatalysts. J. Mater. Chem..

[9] An H., Du Y., Wang T., Wang C., Hao W., Zhang J. (2008). Photocatalytic properties of BiOX (X = Cl, Br, and I). Rare Met..

[10] Zhang X., Ai Z., Jia F., Zhang L. (2008). Generalized One-Pot Synthesis, Characterization, and Photocatalytic Activity of Hierarchical BiOX (X = Cl, Br, I) Nanoplate Microspheres. J. Phys. Chem. C.

[11] Cao J., Luo B., Lin H., Chen S. (2011). Photocatalytic activity of novel AgBr/WO_3_ composite photocatalyst under visible light irradiation for methyl orange degradation. J. Hazard. Mater..

[12] Ye L., Liu J., Jiang Z., Peng T., Zan L. (2013). Facets coupling of BiOBr-g-C_3_N_4_ composite photocatalyst for enhanced visible-light-driven photocatalytic activity. Appl. Catal. B Environ..

[13] Song S., Gao W., Wang X., Li X., Liu D., Xing Y., Zhang H. (2012). Microwave-assisted synthesis of BiOBr/graphene nanocomposites and their enhanced photocatalytic activity. Dalton Trans..

[14] Cao J., Xu B., Luo B., Lin H., Chen S. (2011). Novel BiOI/BiOBr heterojunction photocatalysts with enhanced visible light photocatalytic properties. Catal. Commun..

[15] Siao C.W., Chen H.L., Chen L.W. (2018). Controlled Hydrothermal Synthesis of Bismuth Oxychloride/Bismuth Oxybromide/Bismuth Oxyiodide Composites Exhibiting Visible-Light Photocatalytic Degradation of 2-Hydroxybenzoic Acid and Crystal Violet. J. Colloid Interface Sci..

[16] Shenawi-Khalil S., Uvarov V., Fronton S., Popov I., Sasson Y. (2012). A Novel Heterojunction BiOBr/Bismuth Oxyhydrate Photocatalyst with Highly Enhanced Visible Light Photocatalytic Properties. J. Phys. Chem. C.

[17] Di J., Xia J., Ge Y., Xu L., Xu H., Chen J., He M., Li H. (2014). Facile fabrication and enhanced visible light photocatalytic activity of few-layer MoS_2_ coupled BiOBr microspheres. Dalton Trans..

[18] Xu C., Wu H., Gu F.L. (2014). Efficient adsorption and photocatalytic degradation of Rhodamine B under visible light irradiation over BiOBr/montmorillonite composites. J. Hazard Mater..

[19] Lin H.P., Chen C.C., Lee W.W. (2016). Synthesis of SrFeO_3−x_/g-C_3_N_4_ heterojunction with improved visible-light photocatalytic activities in chloramphenicol and crystal violet degradation. RSC Adv..

[20] Zhang Z., Xu L., Liu C. (2015). Preparation and characterization of composite magnetic photocatalyst MnxZn_1-x_Fe_2_O_4_/β-Bi_2_O_3_. RSC Adv..

[21] Zhang L., Wang W., Zhou L., Shang M., Sun S. (2009). Fe_3_O_4_ coupled BiOCl: A highly efficient magnetic photocatalyst. Appl. Catal. B Environ..

[22] Kong L., Jiang Z., Xiao T., Lu L., Jones M.O., Edwards P.P. (2011). Exceptional visible-light-driven photocatalytic activity over BiOBr-ZnFe_2_O_4_ heterojunctions. Chem. Commun..

[23] Jiang R., Zhu H.Y., Li J.B., Fu F.Q., Yao J., Jiang S.T., Zeng G.M. (2016). Fabrication of novel magnetically separable BiOBr/CoFe_2_O_4_ microspheres and its application in the efficient removal of dye from aqueous phase by an environment-friendly and economical approach. Appl. Surf. Sci..

[24] Xu Y., Zhou T., Huang S., Xie M., Li H., Xu H., Xia J., Li H. (2015). Preparation of magnetic Ag/AgCl/CoFe_2_O_4_ composites with high photocatalytic and antibacterial ability. RSC Adv..

[25] Pullar R.C. (2012). Hexagonal ferrites: A review of the synthesis, properties and applications of hexaferrite ceramics. Prog. Mater. Sci..

[26] Xie T., Liu C., Xu L., Yang J., Zhou W. (2013). Novel Heterojunction Bi_2_O_3_/SrFe_12_O_19_ Magnetic Photocatalyst with Highly Enhanced Photocatalytic Activity. J. Phys. Chem. C.

[27] Xie T., Xu L., Liu C., Yang J., Wang M. (2014). Magnetic composite BiOCl-SrFe_12_O_19_: A novel p-n type heterojunction with enhanced photocatalytic activity. Dalton Trans..

[28] Zhang L.L., Xiong Z., Zhao X.S. (2010). Pillaring Chemically Exfoliated Graphene Oxide with Carbon Nanotubes for Photocatalytic Degradation of Dyes under Visible Light Irradiation. ACS Nano.

[29] Jo W.K., Tayade R.J. (2014). New Generation Energy-Efficient Light Source for Photocatalysis: LEDs for Environmental Applications. Ind. Eng. Chem. Res..

[30] Natarajan K., Bajaj H.C., Tayade R.J. (2016). Photocatalytic efficiency of bismuth oxyhalide (Br, Cl and I) nanoplates for RhB dye degradation under LED irradiation. J. Ind. Eng. Chem..

[31] Arlos M.J., Liang R., Hatat-Fraile M.M., Bragg L.M., Zhou N.Y., Servos M.R., Andrews S.A. (2016). Photocatalytic decomposition of selected estrogens and their estrogenic activity by UV-LED irradiated TiO_2_ immobilized on porous titanium sheets via thermal-chemical oxidation. J. Hazard. Mater..

[32] Kamzin A.S., Lampen-Kelley P., Phan M.H. (2016). Mossbauer and magnetic studies of the phase state of SrFe_12_O_19_/La_0.9_Ca_0.1_MnO_3_ composites. Phys. Solid State.

[33] Lee Y.H., Dai Y.M., Fu J.Y. (2017). A series of bismuth-oxychloride/bismuth-oxyiodide/graphene-oxide nanocomposites: Synthesis, characterization, and photcatalytic activity and mechanism. Mol. Catal..

[34] Jiang Y.R., Lin H.P., Chung W.H. (2015). Controlled hydrothermal synthesis of BiO_x_Cl_y_/BiO_m_I_n_ composites exhibiting visible-light photocatalytic degradation of crystal violet. J. Hazard. Mater..

[35] Lu L., Zhou M.Y., Yin L., Zhou G.W., Jiang T., Wan X.K., Shi H.X. (2016). Tuning the physicochemical property of BiOBr via pH adjustment: Towards an efficient photocatalyst for degradation of bisphenol A. J. Mol. Catal. A Chem..

[36] Pullar R.C. (2012). Combinatorial Bulk Ceramic Magnetoelectric Composite Libraries of Strontium Hexaferrite and Barium Titanate. ACS Comb. Sci..

[37] Cui C., Xu L., Xie T., Peng T. (2016). Synthesis and photocatalytic activity of magnetic heterostructure ZnFe_2_O_4_-SrFe_12_O_19_. Mater. Technol..

[38] Xie T., Xu L., Liu C., Wang Y. (2013). Magnetic composite ZnFe_2_O_4_/SrFe_12_O_19_: Preparation, characterization, and photocatalytic activity under visible light. Appl. Surf. Sci..

[39] Das M., Bhattacharyy K.G. (2014). Oxidation of Rhodamine B in aqueous medium in ambient conditions with raw and acid-activated MnO_2_, NiO, ZnO as catalysts. J. Mol. Catal. A Chem..

[40] Xu H., Li H.M., Sun G.S., Xia J.X., Wu C.D., Ye Z.X., Zhang Q. (2010). Photocatalytic activity of La_2_O_3_-modified silver vanadates catalyst for Rhodamine B dye degradation under visible light irradiation. Chem. Eng. J..

[41] Cui Y.F., Goldup S.M., Dunn S. (2015). Photodegradation of Rhodamine B over Ag modified ferroelectric BaTiO_3_ under simulated solar light: Pathways and mechanism. RSC Adv..

