# Supplementary material for: Visible-Light-Driven Photocatalytic Activity of Magnetic BiOBr/SrFe12O19 Nanosheets
Source: Nanomaterials (Basel). 2019 May 13;9(5):735. doi: 10.3390/nano9050735 (PMC6567020; doi:10.3390/nano9050735)
Supplement: Supplementary file 1 [file nanomaterials-09-00735-s001.pdf]

# Supplementary Materials

## Visible-Light-Driven Photocatalytic Activity of Magnetic $BiOBr/SrFe_{12}O_{19}$ Nanosheets

**Taiping Xie [1,2], Jiao Hu [3], Jun Yang [4], Chenglun Liu [1,3,*], Longjun Xu [1,*], Jiankang Wang [2], Yuan Peng [2], Songli Liu [2,*], Xiuyu Yin [3] and Yuanzhen Lu [3]**

1 State Key Laboratory of Coal Mine Disaster Dynamics and Control, Chongqing University, Chongqing 400044, China; deartaiping@163.com (T.X.)

2 Chongqing Key Laboratory of Extraordinary Bond Engineering and Advanced Materials Technology (EBEAM), Yangtze Normal University, Chongqing 408100, China; wjkwjk074478@163.com (J.W.); pengyuan1030@sina.com (Y.P.)

3 College of Chemistry and Chemical Engineering, Chongqing University, Chongqing 401331, China; (hujiao666@163.com) (J.H.); xinyuyin@163.com (X.Y.); yuanzhenlu@163.com (Y.L.）

4 College of Materials and Chemical Engineering, Chongqing University of Arts and Sciences, Yongchuan 402160, China; yang_jun_yang_jun@163.com(J.Y.)

* Correspondence: xlclj@cqu.edu.cn (C.L.); xulj@cqu.edu.cn (L.X.); pzhlsll@163.com (S.L.)

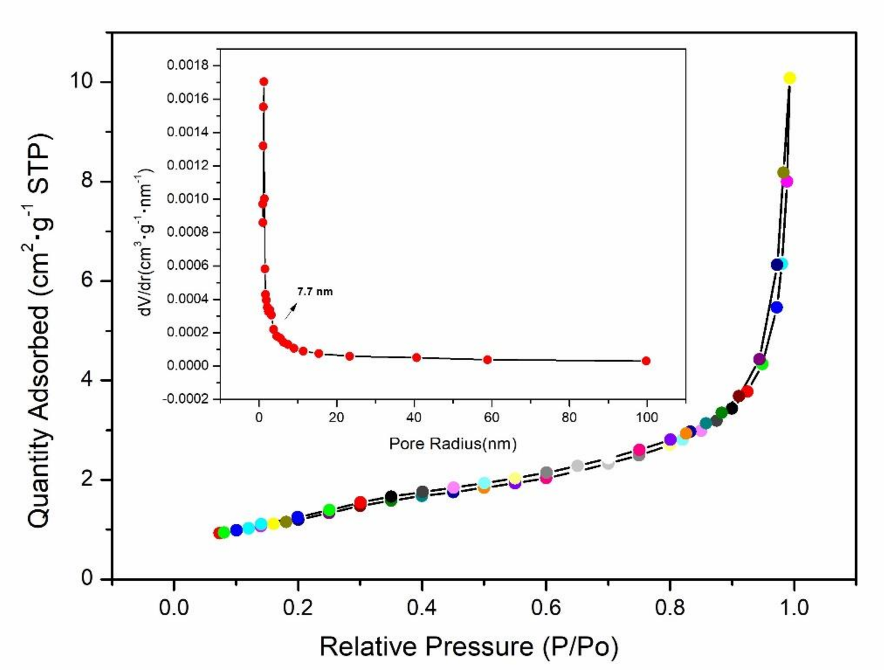


**Figure S1.** Adsorption–desorption isotherms and the pore size distribution curves (Inset) for BOB/SOF-5.

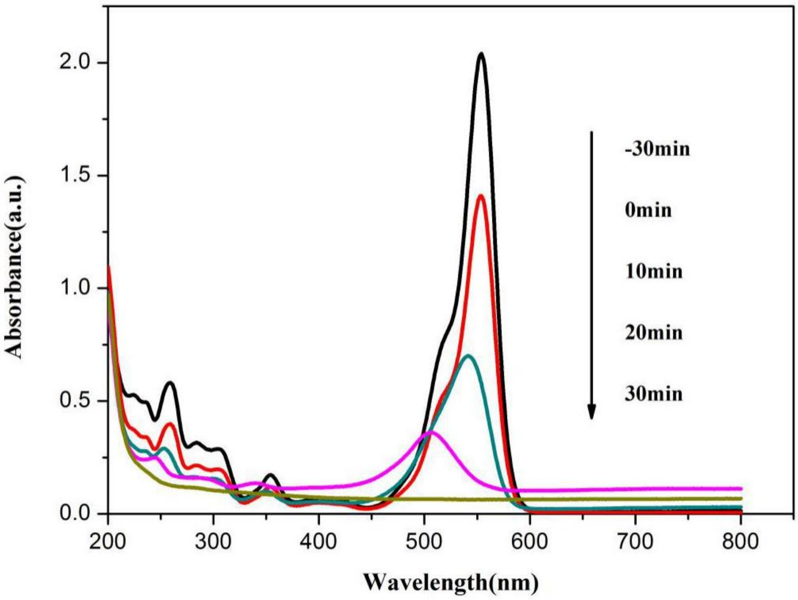


**Figure S2.** Absorption curves of RhB with BOB/SFO-5 under visible light irradiation.

**Table S1.** The estimated Eg values of the as-prepared samples.

| Samples | The Estimated Eg Value (eV) |
|---|---|
| BiOBr | 2.80 |
| BOB/SFO-3 | 2.70 |
| BOB/SFO-5 | 2.67 |
| BOB/SFO-7 | 2.63 |
| BOB/SFO-10 | 2.60 |
| BOB/SFO-15 | 2.58 |

**Table S2.** Magnetic parameters of the as-synthesized samples.

| Samples | Saturation Magnetization (Ms, emu·g$^{-1}$) | Remanent Magnetization (Mr, emu·g$^{-1}$) | Coercivity (Hc, G) |
|---|---|---|---|
| $SrFe_{12}O_{19}$ | 38.95 | 17.02 | 1216.66 |
| BOB/SFO-5 | 4.39 | 1.11 | 861.04 |

**Table S3.** Comparison of photodegradation ratio using different photocatalyst under visible light irradiation reported in the past ten years.

| Photocatalysts | Photodegradation Ratio | Photodegradation Reaction Time (min) | Refs. |
|---|---|---|---|
| BOB/SFO-5 | 97.0% | 30 | This work |
| $Ag_3PO_4$ nanoparticles | 80.50% | 45 | [1] |
| g-$C_3N_4$ with sacrificial KIT-6 template | 100% | 50 | [2] |
| P-doped g-$C_3N_4$ | 100% | 50 | [3] |
| Copper fiber@ZnO/CdS | 90% | 60 | [4] |
| Square-sharped BiOCl nanosheets | 98% | 60 | [5] |
| 2D $MoS_2$/Red phosphorus heterojunction | 97.50% | 80 | [6] |
| Zero Valent $Bi^{(0)}$ incorporated bismuth terephthalate | 97% | 80 | [7] |
| CdS/Ag/a-$TiO_2$ | 82% | 80 | [8] |
| MIL-88A(Fe)/grapheme oxide composite | 100% | 100 | [9] |
| Zero Valent $Fe^{(0)}$ doped g-$C_3N_4$/$MoS_2$ | 98.20% | 150 | [10] |
| Hexagonal/monoclinic-$WO_3$ | 91% | 180 | [11] |
| Fluorinated $Bi_2WO_6$ | 98% | 210 | [12] |
| $TiO_2$ with interface defects | 75% | 300 | [13] |


[1] RSC Adv., 7 (2017) 40896–40904.
[2] Appl. Surf. Sci., 396 (2017) 78–84.
[3] ACS Sustainable Chem. Eng., 6 (2018) 6342–6349.
[4] ACS Sustainable Chem. Eng., 6 (2018) 155–164.
[5] Appl. Surf. Sci., 439 (2018) 697–704.
[6] Mater. Lett., 222 (2018) 187–191.
[7] J. Phys. Chem. Solids, 111 (2017) 431–438.
[8] RSC Adv., 8(2018) 13625–13634.
[9] Appl. Catal., B, 221(2018) 119–128.
[10] ACS Sustainable Chem. Eng., 4(2016) 4055–4063.
[11] Energy Mater., 1(2018) 2067–2077.
[12] Environ. Sci. Technol., 42(2008) 2085–2091.
[13] Langmuir, 26(2010) 9686–9694.


It was worth mentioning that the photocatalytic activity of BOB/SFO-5 for Rhodamine B (RhB) photodegradation was very outstanding. The photodegradation ratio of RhB could reach to 97.0% after only 30 min photocatalytic reaction under visible light irradiation. The photocatalytic efficiency was superior to that of the existing literature reports.
